# Psychometric Evaluation of the Identification with the Country Scale in a Chilean Sample

**DOI:** 10.3390/bs16071106

**Published:** 2026-07-03

**Authors:** Rodrigo Landabur, Carlos Escobar-Campusano, Crhistian Rojo, Almendra Pereira, Jorge Flores-Torres

**Affiliations:** 1 Departamento de Psicología, Universidad de Atacama, Copiapó 1531772, Chile; rodrigo.landabur@uda.cl (R.L.); crhistian.rojo.24@alumnos.uda.cl (C.R.); almendra.pereira.24@alumnos.uda.cl (A.P.); 2 Departamento de Personalidad, Evaluación y Tratamiento Psicológico, Facultad de Psicología y Logopedia, Universidad de Málaga, 29071 Málaga, Spain; jorgeflorestorres@uma.es

**Keywords:** group identification, social identity theory, factor structure, identity fusion

## Abstract

Group identification refers to a psychological connection with a group in which individuals incorporate group-defining characteristics into their self-concept. The scale developed by Mael and Ashforth is one of the most used, but it has not been examined in Chile. This study analyzed the psychometric properties of this scale in a Chilean sample using the country as the reference group. A one-factor structure and convergent validity were expected. The one-factor fit of the scale was evaluated in a non-probability sample (n = 523) through confirmatory factor analysis. The results were consistent with an essentially unidimensional structure (χ^2^/df = 3.99, *p* < 0.001, CFI = 0.973, TLI = 0.955, SRMR = 0.032, and RMSEA = 0.091), but they must be taken with caution. The model presented adequate factor loadings (>0.500), a high reliability (α = 0.87 and ω = 0.87) and convergent validity (identification with Chile and identity fusion with Chile measurements are related, *r* = 0.46–0.48, *p* < 0.001), although they represent different constructs. Finally, the model showed invariance for gender. The scale’s relevance was discussed according to the possible positive and negative effects of identification with the country, particularly in contexts with important migration processes.

## 1. Introduction

Social identity theory is a foundational framework in social psychology that seeks to explain how people behave in terms of their social identities (Ellemers & Haslam, 2012). This theory posits that to enhance self-esteem, individuals categorize themselves and others into social groups (Tajfel, 1974; Tajfel & Turner, 1979; Turner & Reynolds, 2012). Subsequent research has suggested that self-esteem is not the sole reason for categorization (Ellemers & Haslam, 2012). Social identity theory further outlines the psychological dynamics involved in the distinction between personal and social identity, the strategies individuals use to achieve a positive social identity, and the structural characteristics of the social context that influence which strategies are more likely to be adopted in a given situation (Ellemers & Haslam, 2012).

Personal identity refers to a part of the self-concept composed of idiosyncratic characteristics (e.g., individual abilities), whereas social identity denotes the component of the self-concept derived from group membership and the emotional significance attached to that membership (Tajfel, 1974; Turner & Reynolds, 2012). Group identification is a central concept in social identity theory that links personal and social identities. This refers to a psychological connection with a group whereby individuals incorporate group-defining characteristics into their self-concept, such that social identity becomes salient and predominates over personal identity (Mael & Ashforth, 1992; Riketta, 2005; Tajfel & Turner, 1979). Within this framework, personal and social identities are often conceptualized as operating hydraulically (i.e., when one identity is salient, the other recedes; Turner & Reynolds, 2012). Similarly, participants tend to perceive group members who embody prototypical characteristics as more relevant, whereas other members are viewed as interchangeable (Swann et al., 2012; Yzerbyt & Demoulin, 2010). The identification strength with a given group is dynamic and may vary over time and across social contexts (Doosje et al., 2002). 

Group identification is influenced by group status. People who define themselves as members of a high-status group are more likely to achieve a positive social identity than members of low-status groups (Ellemers, 1993). When such distinctiveness is an integral part of an individual’s identity (Spiteri et al., 2026), high group status is associated with admiration among its members, which in turn enhances self-esteem (Mael & Ashforth, 2001).

Group identification is grounded in the need of individuals to align their sense of self with a collective (Ashforth & Mael, 2024). For example, group identification in an organizational context involves feeling pride in group membership and internalizing the group’s values (Riketta, 2005). Consequently, belonging to a group shapes individuals’ attitudes and behavior, particularly when they define themselves in terms of their group membership (Van Knippenberg & Van Schie, 2000). Accordingly, group identification promotes a range of intragroup behaviors that strengthen the group (Tajfel & Turner, 1979; Varmann et al., 2023; Zhang et al., 2022), such as increased work engagement (Rongzhi & Qing, 2026), organizational commitment (Van Knippenberg & Sleebos, 2006), reduced turnover intentions (Basar & Filizoz, 2015), and greater group cohesion, as observed among active runners (Stevens et al., 2019) and police officers and firefighters (García-Guiu López et al., 2015). In addition, group identification is associated with positive outcomes beyond the group context, including work satisfaction across occupations (Lee et al., 2015; Ng, 2015; Van Knippenberg & Van Schie, 2000), lower levels of teachers’ burnout (Uzun, 2018), and increased engagement in collective action to support individuals with disabilities (Pérez-Garín et al., 2021). Identification with the country can be linked to both positive and negative outcomes. On the one hand, it has been associated with increased well-being over time (Khan et al., 2020) and adaptive responses in stressful contexts such as the COVID-19 pandemic (Bonetto et al., 2022). However, it may also be associated with negative consequences, including prejudice among locals toward immigrants (Landabur et al., 2024) and reciprocal prejudice among immigrant populations (González et al., 2010).

### 1.1. Group Identification Measurement

Several instruments, most of which are Likert-type scales, have been developed to assess group identification, which measures individuals’ perceived connection with a specific group or social entity. In the present study, we focused on the scale developed by Mael and Ashforth because it is one of the most widely used measures of group identification (Riketta, 2005) and has demonstrated superior predictive validity for extreme pro-group behaviors compared with other prominent identification measures (e.g., Ellemers et al., 1999; Leach et al., 2008; Postmes et al., 2013; Steffens et al., 2015; see Varmann et al., 2023 for a review). Although originally developed in organizational settings, the scale has been successfully applied to a wide range of targets, including national identity (Gómez et al., 2011), religious groups (Besta et al., 2014), and even non-social entities such as store brands (Rubio et al., 2015).

The Mael & Ashforth scale is a self-report Likert-type instrument that consistently exhibits a unidimensional structure across diverse cultural contexts, including samples from the United States (Mael & Ashforth, 1992), Turkey (Basar & Filizoz, 2015; Öz & Meriç, 2026), and Romania (Boroş, 2008). Across studies, the scale has demonstrated acceptable internal consistency (α > 0.70; Contreras-Pacheco & Lesmez-Peralta, 2018; García-Guiu López et al., 2015; Irshad & Bashir, 2020; Pérez-Garín et al., 2021; Rodríguez-Carvajal et al., 2014; Van Knippenberg & Van Schie, 2000), and factor loadings have generally exceeded 0.50 in samples from the United States, Spain, Turkey, Colombia, and Romania (Anaza, 2015; Basar & Filizoz, 2015; Boroş, 2008; Contreras-Pacheco & Lesmez-Peralta, 2018; García-Guiu López et al., 2015; Gómez et al., 2011; Öz & Meriç, 2026).

### 1.2. This Study

The Mael and Ashforth scale has been applied to various referent targets, including organizations, social groups, and abstract entities. This study focuses on identifying the target country. Although this construct has been extensively examined in developed societies, it has received less attention in Latin American contexts. To the best of our knowledge, the psychometric properties of the Mael and Ashforth scale have not yet been examined in a Chilean sample. Accordingly, this study aims to evaluate the psychometric properties of the Mael and Ashforth scale in a Chilean sample. Chile is particularly interesting given its unique characteristics: it is geographically isolated and culturally homogeneous, though high levels of immigration have recently challenged the latter. Thus, evaluating the Mael and Ashforth scale in this society provides a test of the country’s cross-cultural stability.

We propose the following hypotheses:

**H1.**  *The scale will show structural validity, consisting of a one-factor model with acceptable factor loadings (>0.500) and fit indexes (χ^2^/df < 5; CFI > 0.95; TLI > 0.95; RMSEA < 0.08, and SRMR < 0.08).*

**H2.**  *Identification with Chile will show adequate reliability (α > 0.70 and ω > 0.70).*

Convergent validity was assessed using identity fusion, a construct that captures a strong relational bond with a group. Identity fusion refers to a visceral sense or feeling of oneness with a group that motivates pro-group behaviors, including a willingness to engage in extreme actions or personal sacrifice for the benefit of other group members (Gómez et al., 2020; Swann et al., 2015). A substantial body of research has shown that identity fusion robustly explains pro-group behaviors (Buhrmester et al., 2014; Gómez et al., 2011; Swann et al., 2014a; Swann et al., 2010; Swann et al., 2012; Whitehouse et al., 2014) and differs from traditional group identification in several key respects. Identity fusion is characterized by identity synergy, in which personal and social identities are functionally equivalent and mutually reinforcing. In contrast, group identification models conceptualize these identities as compensatory or “hydraulic” (Turner & Reynolds, 2012). Second, identity fusion involves strong relational ties, fostering deep, familial-like bonds among group members, who are valued for their unique personal qualities. In contrast, group identification emphasizes prototypicality, in which individuals who best represent the group’s defining characteristics are highly valued (Swann et al., 2012; Yzerbyt & Demoulin, 2010). Third, identity fusion tends to be more stable and enduring over time than group identification, which is more context-dependent and variable (Gómez et al., 2020).

Although identification with the country and identity fusion with the country are positively associated (0.29 < *r* < 0.88; Besta et al., 2014; Gómez et al., 2011), factor-analytic evidence indicates that they are empirically distinct constructs (Gómez et al., 2011). Accordingly, we hypothesized:

**H3.**  *Identification scale scores are positively associated with identity fusion.*

This study provides evidence for the psychometric properties of the scale, a necessary condition for its rigorous application in future research, in which group identification may play a central explanatory role. This is especially relevant in Chile because previous studies have related identification with Chile with the following variables: perceived symbolic threat toward specific immigrant communities (Venezuelans but not Peruvians; Landabur et al., 2024); negative emotional reactions in Chileans aligned with a left (but not right) political position in evaluating their perception toward a past dictatorship (González et al., 2013); and it is an important base for social cohesion (Consejo de Cohesión Social, 2020).

## 2. Materials and Methods

This study employed a cross-sectional instrumental design (Ato et al., 2013). The psychometric properties of Mael and Ashforth’s group identification scale were examined in a Chilean sample. This study was conducted as part of a broader research project aimed at evaluating the effect of identity fusion with the country in predicting prejudice toward immigrants.

### 2.1. Participants

A non-probability sample of 581 Chileans was recruited. Subjects were excluded because they were not Chileans (24), their ages were not between 18 and 59 years (18), and they were not from two specific geographical places (Atacama and Metropolitan regions, 16); these exclusion criteria were associated with the broader project in which this study was included. The age range was used to prevent overexposure in older adults at retirement age, a subpopulation considered as potentially vulnerable. Both regions were selected because the authors reside in Atacama, and the capital of the country is located in the Metropolitan region. The final sample comprised 523 Chilean participants (age range: 18–52 years; M = 22.8, SD = 6.99; 61.76% were women). Participants reported diverse occupational positions, including students (61.76%), students and workers (26.00%), and workers (12.24%). Monte Carlo simulations showed that this sample size, with an error type 1 of 0.05, allows for detecting factor loadings of >0.500 with a power of >0.99, which is higher than the recommended value of 0.80.

### 2.2. Instruments

#### 2.2.1. Identification with the Country

We used a back translation procedure (Berry, 1980) to adapt the group identification scale developed by Mael and Ashforth (1992) to the Chilean Spanish context, replacing references to “school” (or the specific institution) with “my country”. Participants indicated their agreement with six statements assessing their relationship with their country on a Likert-type scale ranging from 0 (Totally disagree) to 4 (Totally agree). Examples include “When someone criticizes my country, it feels like a personal insult” and “The successes of my country are my successes”. Previous studies have reported good reliability for this scale (α between 0.74 and 0.90; García-Guiu López et al., 2015; Gómez et al., 2011; Jones & Volpe, 2011; Rongzhi & Qing, 2026; Ozturk & Soyturk, 2021).

#### 2.2.2. Identity Fusion with the Country

Identity fusion was assessed using the verbal fusion scale’s Spanish version (Gómez et al., 2011). Participants rated their agreement with seven items describing their relationship with their country on a scale ranging from 0 (strongly disagree) to 6 (strongly agree). Example items include “I am one with my country,” and “I have a deep emotional bond with my country.” This scale has shown a two-factor structure in Latin American samples, including Chile (Henríquez et al., 2019; Landabur et al., 2022): feelings of connectedness (an example item is “I am one with my country”) and reciprocal strength (e.g., “I am strong because of my country”). This scale has presented adequate reliability: αs ranging from 0.70 to 0.95 (Besta et al., 2014; Besta et al., 2015; Buhrmester et al., 2014; Gómez et al., 2011; Landabur et al., 2022; Swann et al., 2014a; Swann et al., 2014b). In this study, reliability was very high for the total scale (α = 0.92 and ω = 0.92) and high for the feelings of connectedness (α = 0.86 and ω = 0.86) and reciprocal strength (α = 0.88 and ω = 0.88) components.

### 2.3. Procedure

Participants were recruited through an online survey distributed on social media (e.g., Facebook and Instagram) and professional networking (e.g., LinkedIn) platforms, institutional e-mail lists from Chilean universities, and personal networks. Recipients were also encouraged to share the survey with their personal contacts, thereby increasing the sample’s reach.

The invitation included a link to the study, which participants accessed voluntarily. Upon accessing the survey, the participants electronically provided informed consent before proceeding. The participants then completed the identity fusion and group identification scales and provided their demographic information. The survey required approximately four minutes to complete. Responses with missing data were excluded from the analysis.

### 2.4. Data Analysis

A confirmatory factor analysis was conducted to evaluate the scale’s factor structure and assess reliability using Cronbach’s α and McDonald’s ω. Factor loadings greater than 0.500 were considered acceptable (J. Hair et al., 1999). Given the sensitivity of the χ^2^ statistic to sample size, well-fitting models are often rejected when *N* > 200 (J. Hair et al., 1999; Ruiz et al., 2010). Model fit was evaluated using multiple indices. Specifically, acceptable model fit was defined as *χ*^2^/df < 5 (Kline, 2016), TLI > 0.95, CFI > 0.95, RMSEA < 0.08, and SRMR < 0.08 (Mîndrilă, 2010; Schreiber, 2017). The common variance explained by the latent factor is acceptable if > 50%, because it is the value usually found in social sciences (J. F. Hair et al., 2014). Reliability was interpreted according to the following criteria: very low (α < 0.30), low (0.30 < α ≤ 0.60), moderate (0.60 < α ≤ 0.75), high (0.75 < α ≤ 0.90), and very high (α > 0.90; Alexandre et al., 2013).

The correlation between the identification scale and the identity fusion measure was used to assess convergent validity. Additionally, we evaluated discriminant validity by comparing measurement models, considering differences to be meaningful when |ΔCFI| > 0.010 (Cheung & Rensvold, 2002). Measurement invariance across gender was also examined (coded as 0 for women and 1 for men). Configural, metric, and scalar invariance models were tested sequentially. Following Chen (2007), invariance was supported when changes in fit indices met the following criteria: for metric invariance, |ΔCFI| < 0.010, |ΔRMSEA| < 0.015, and |ΔSRMR| < 0.030; for scalar and strict invariance, |ΔCFI| < 0.010, |ΔRMSEA| < 0.015, and |ΔSRMR| < 0.010. All analyses were performed using RStudio (Version 4.2) and the Lavaan (Version 0.6-21) Package (Rosseel, 2012).

### 2.5. Ethical Considerations

This study was conducted as part of a broader project examining prejudice toward outgroups. The project was approved by the Ethical Committee of the Universidad de Atacama, adhering to institutional guidelines and the principles of the Declaration of Helsinki. Participation was voluntary; all participants provided their informed consent electronically before completing the survey and then responded to the scales detailed above.

## 3. Results

Table 1 presents the descriptive statistics and interitem correlations. Multivariate normality was assessed using Mardia’s test (Mardia, 1974), which indicated significant deviations from normality (skewness: *b* = 2.44, *p* < 0.001; kurtosis: *b* = 57.14, *p* < 0.001). Accordingly, Spearman’s rank-order correlations were computed, and confirmatory factor analysis was conducted using Maximum Likelihood with Robust standard errors (MLR) as the estimator, which is appropriate for non-normal data (Muthén & Muthén, 1998–2017). All items were positively and significantly correlated (*p* < 0.001).

The fit indexes for the unidimensional model were: χ^2^/df = 35.910/9 = 3.99 (χ^2^ was significant at *p* < 0.001), CFI = 0.973, TLI = 0.955, RMSEA = 0.091, and SRMR = 0.032. All factor loadings were >0.500 (see Table 2), and the proportion of explained variance by the model was 53.22%. In addition, the scale demonstrated high reliability, α = 0.87 and ω = 0.87.

Convergent validity was examined by assessing the relationship between identification with Chile and identity fusion with Chile. The last scale showed an essentially two-factor structure (χ^2^/df = 3.87, CFI = 0.979, TLI = 0.962, RMSEA = 0.092, and SRMR = 0.030, in which a covariance was included among the items “I am one with my country” and “My country is me” because they are similarly redacted) with adequate factor loadings (>0.500). As expected, identification and identity fusion were positively related to the feelings of connectedness (*r* = 0.46, *p* < 0.001), and reciprocal strength (*r* = 0.48, *p* < 0.001) factors. We evaluated their distinctiveness as an additional discriminant validity analysis by comparing a one-factor model with a three-factor model. The three-factor model showed a better fit to the data (χ^2^/df = 3.18, CFI = 0.962, TLI = 0.951, RMSEA = 0.071, and SRMR = 0.046) than the one-factor model (χ^2^/df = 14.16, CFI = 0.753, TLI = 0.700, RMSEA = 0.175, and SRMR = 0.118), with the difference in fit indices exceeding the recommended threshold |ΔCFI| > 0.010.

Finally, we evaluated measurement invariance across gender (see Table 3). The configural model demonstrated an acceptable fit (CFI > 0.95, TLI > 0.95, RMSEA < 0.08, and SRMR < 0.08), indicating a similar factor structure across groups. Subsequent comparisons showed that the differences between configural and metric models, metric and scalar models, and scalar and strict models met the recommended criteria |ΔCFI| < 0.010 and |ΔRMSEA| < 0.015, and particularly, |ΔSRMR| < 0.030 for metric, and |ΔSRMR| < 0.010 for scalar and strict models.

## 4. Discussion

This study examined the psychometric properties of identification with the country scale developed by Mael and Ashforth (1992) in a Chilean sample. Fit indices were generally adequate, except for RMSEA, which slightly exceeded the conventional cut-off (0.08). Furthermore, factor loadings and reliability were acceptable, and the scale showed convergent validity and invariance across gender. The common variance explained (53%) suggests that the general factor accounts for a substantial proportion of the common variance among the items. Taken together, our results provide evidence that is consistent with an essentially unidimensional structure and not an unequivocally acceptable model fit. Although the RMSEA tends to be artificially inflated in models with low degrees of freedom and may overestimate model misfit in such cases (Kenny et al., 2015), this explanation does not by itself rule out a potential model misfit. Overall, these findings add to the extensive body of literature supporting the validity of this group identification scale across samples from different countries (e.g., Basar & Filizoz, 2015; Boroş, 2008; Rongzhi & Qing, 2026; Mael & Ashforth, 1992).

Assessing identification with the country may be particularly relevant in contexts such as Chile, as it can help to better predict a range of social and psychosocial outcomes. On the one hand, challenges related to quality of life may be mitigated by the increased well-being associated with national identification (Khan et al., 2020), especially in adverse scenarios (Bonetto et al., 2022). However, group identification may also be associated with negative outcomes. For example, studies in Chile have reported prejudice among locals toward immigrants (Landabur et al., 2024) as well as prejudice among immigrants toward natives (González et al., 2010). This issue is particularly important given that the country has experienced substantial increases in immigration in recent years (Instituto Nacional de Estadísticas & Servicio Nacional de Migraciones, 2024).

The unidimensional structure, adequate reliability (>0.70), and factor loadings (>0.500) observed in this study are consistent with those reported in previous studies (e.g., Anaza, 2015; Basar & Filizoz, 2015; Contreras-Pacheco & Lesmez-Peralta, 2018; García-Guiu López et al., 2015; Gómez et al., 2011; Jones & Volpe, 2011; Mael & Ashforth, 1992; Rongzhi & Qing, 2026). The consistency of these findings across studies suggests that the Mael and Ashforth scale is a robust instrument that remains stable across different cultural contexts. Future research could extend these findings by examining predictive and longitudinal validity, which, to the best of our knowledge, has not yet been explored in Chile.

Identification with the country was positively associated with identity fusion with the country, supporting the scale’s convergent validity and aligning with established literature (Besta et al., 2014; Gómez et al., 2011; Swann et al., 2010). Additionally, factor analysis indicated that these constructs are different, consistent with prior findings (Gómez et al., 2011). This pattern may be explained by the fact that both constructs reflect forms of group bonding, yet differ in their underlying properties, as previously explained (for a review, see Gómez et al., 2020). For example, while identification emphasizes social identity over personal identity, identity fusion conceptualizes both identities as functioning together (Turner & Reynolds, 2012).

Measurement invariance analysis demonstrated that the scale functions equally across men and women. Thus, the instrument appears to assess the construct in a comparable manner across gender groups. This finding supports the use of this scale for cross-gender comparisons while reducing concerns that observed differences would be attributable to differential measurement functioning, rather than genuine differences in group identification. However, it does not entirely rule out other alternative explanations or sources of bias.

In this study, we used MLR as the CFA estimator. However, a methodological debate exists regarding the treatment of Likert-type items in CFA, which depends on factors such as the number of response categories, sample size, threshold distributions, non-normality, and model complexity. Following Rhemtulla et al. (2012), in Likert items with 5 or more response categories (as in our case), MLR and robust categorical least squares (cat-LS) both estimate values within an acceptable range of bias and show similar rates of Type I error. We used MLR because it provides robust standard errors and a chi-square test statistic that is corrected for departures from multivariate normality with a relatively large sample size (N = 523) and five response categories. In contrast to MLR, cat-LS tends to overestimate parameters under a non-normal distribution (Rhemtulla et al., 2012).

The limitations of this study relate to sampling, sample composition, and the choice of reference group. First, a non-probabilistic sampling technique was used. Second, the sample was predominantly young and largely composed of students from only two geographical regions in Chile. Both characteristics limit the generalizability of the results to the broader Chilean population, thereby limiting the study’s overall external validity. Specifically, older adults or non-student populations may exhibit distinct identity processes, which are influenced by educational context and life experiences. Therefore, future research should replicate our findings in more representative samples to enhance external validity. Third, the psychometric properties of the scale were assessed using the country as the reference group; thus, the results may not be generalizable to other collective targets. This is consistent with the notion that social identification is not a stable dispositional trait (Weber et al., 2011) but rather depends on contextual factors such as group distinctiveness (Grant & Hogg, 2012) and entitativity (Jans et al., 2011). Despite these limitations, the present findings provide initial evidence supporting the reliability and validity of the identification with the country scale in a Chilean context. These results contribute to the cross-cultural literature on group identification and highlight the importance of considering its positive and negative implications. Future research should continue to examine this construct across diverse populations and contexts to further refine its theoretical and empirical understanding.

## Figures and Tables

**Table 1:** Descriptive statistics and correlations among items.

	Mean (*SD*)	Correlations				
		1	2	3	4	5
Item 1. When someone criticizes my country, it feels like a personal insult.	2.21 (1.39)					
Item 2. I am very interested in what citizens of other countries think about my country.	1.80 (1.35)	0.50				
Item 3. When I talk about my country, I usually say “we” rather than “they.”	2.68 (1.35)	0.45	0.40			
Item 4. The successes of my country are my successes.	1.97 (1.37)	0.60	0.45	0.57		
Item 5. When someone praises my country, it feels like a personal compliment.	2.13 (1.40)	0.68	0.49	0.52	0.74	
Item 6. If a story in the media criticized my country, I would feel embarrassed.	1.97 (1.36)	0.49	0.44	0.45	0.48	0.56

**Table 2:** Factor loadings of the identification with Chile scale items.

Item	Factor Loading	*R* ^2^
1	0.766 ***	0.586
2	0.591 ***	0.349
3	0.646 ***	0.418
4	0.817 ***	0.667
5	0.877 ***	0.770
6	0.635 ***	0.404

*Note*. Common variance explained by the latent factor = 53.22% (the sum of the squared standardized factor loadings divided by the number of items). *** *p* < 0.001.

**Table 3:** Configurational, metric, scalar, and strict models for gender invariance.

Model	CFI	ΔCFI	TLI	RMSEA	ΔRMSEA	SRMR	|ΔSRMR|
Configural	0.975		0.959	0.071		0.030	
Metric	0.966	0.009	0.956	0.073	0.002	0.055	0.025
Scalar	0.962	0.004	0.959	0.070	0.003	0.059	0.004
Strict	0.957	0.005	0.962	0.068	0.002	0.055	0.004

## Data Availability

Data are available upon request to the authors.
